# Synthesis and Biological Evaluation of Thio-Derivatives of 2-Hydroxy-1,4-Naphthoquinone (Lawsone) as Novel Antiplatelet Agents

**DOI:** 10.3389/fchem.2020.00533

**Published:** 2020-08-04

**Authors:** Matías Monroy-Cárdenas, Diego Méndez, Andrés Trostchansky, Maximiliano Martínez-Cifuentes, Ramiro Araya-Maturana, Eduardo Fuentes

**Affiliations:** ^1^Instituto de Química de Recursos Naturales, Programa de Investigación Asociativa en Cáncer Gástrico (PIA-CG), Universidad de Talca, Talca, Chile; ^2^Department of Clinical Biochemistry and Immunohaematology, Thrombosis Research Center, Medical Technology School, Faculty of Health Sciences, Universidad de Talca, Talca, Chile; ^3^Departamento de Bioquimica and Center for Free Radical and Biomedical Research, Facultad de Medicina, Universidad de la República, Montevideo, Uruguay; ^4^Centro Integrativo de Biología y Química Aplicada (CIBQA), Escuela de Tecnología Médica, Facultad de Salud, Universidad Bernardo O'Higgins, Santiago, Chile

**Keywords:** lawsone, naphthoquinone, small compounds, platelet, thrombosis

## Abstract

We designed and synthesized in water, using conventional heating and microwave irradiation, new thio-derivatives of 2-hydroxy-1,4-naphthoquinone, a naturally occurring pigment known as lawsone or hennotannic acid, thus improving their antiplatelet activity with relevance to their potential future use in thrombus formation treatment. The structure-activity relationship showed that the thiophenyl moiety enhances the antiplatelet activity. Moreover, the position and nature of the substituent at the phenyl ring have a key effect on the observed biological activity. Compound **4** (2-((4-bromophenyl)thio)-3-hydroxynaphthalene-1,4-dione) was the most active derivative, presenting IC_50_ values for platelet aggregation inhibition of 15.03 ± 1.52 μM for TRAP-6, and 5.58 ± 1.01 μM for collagen. Importantly, no cytotoxicity was observed. Finally, we discussed the structure-activity relationships of these new lawsone thio-derivatives on inhibition of TRAP-6- and collagen-induced platelet aggregation.

## Introduction

Cardiovascular diseases (CVD), one of the leading causes of deaths from non-communicable disease in the world, all have thrombus formation as a common process, with platelet activation at the dysfunctional vessel wall being one of the main steps during thrombosis (ISTH Steering Committee for World Thrombosis Day, 2014; Diamond, 2016).

Platelets are fragments of megakaryocytes circulating within the bloodstream aiding in maintaining hemostasis with both blood components and endothelial cells (Paes et al., 2019). Although platelets have a normal function in primary hemostasis, increased platelet activation has been widely recognized as a key player in the initiation of thrombotic events (Franco et al., 2015). An increase in reactive oxygen species (ROS), [e.g., superoxide and hydrogen peroxide production at the mitochondrial electron transport chain] has been associated with platelet activation (Geisler et al., 2007; Becatti et al., 2013; Caruso et al., 2015). On the other hand, it has been observed that some quinone and hydroquinone compounds protect mitochondrial damage and platelet activation generated by ROS (Kim et al., 2001; Cocheme et al., 2007); in this way, some structure-activity relationship studies with quinone derivatives have been reported (Chang et al., 1997; Cho et al., 1997; Kim et al., 2001). However, given the variability of the mechanisms of action of quinones-hydroquinones it has been suggested that there is a low selectivity on their antiplatelet activity (Bolibrukh et al., 2015; Chang et al., 2019; Mendez et al., 2020). Recent work has shown conflicting results (Fuentes et al., 2018). In this way, 3'-methoxyflavone quinone inhibited arachidonic acid-induced platelet aggregation at low micromolar concentration with an IC_50_ of ~ 10 μM (Chang et al., 2000). Moreover, the presence of OH groups in addition to their methylation or glycosylation in anthraquinones increases the antiplatelet activity (Yun-Choi et al., 1990; Xu et al., 2014). The available drugs with therapeutic ability to regulate platelet activation are still scarce, thus this research area is the high relevance (Mousa, 1999; Moura et al., 2016).

**Figure 1:**
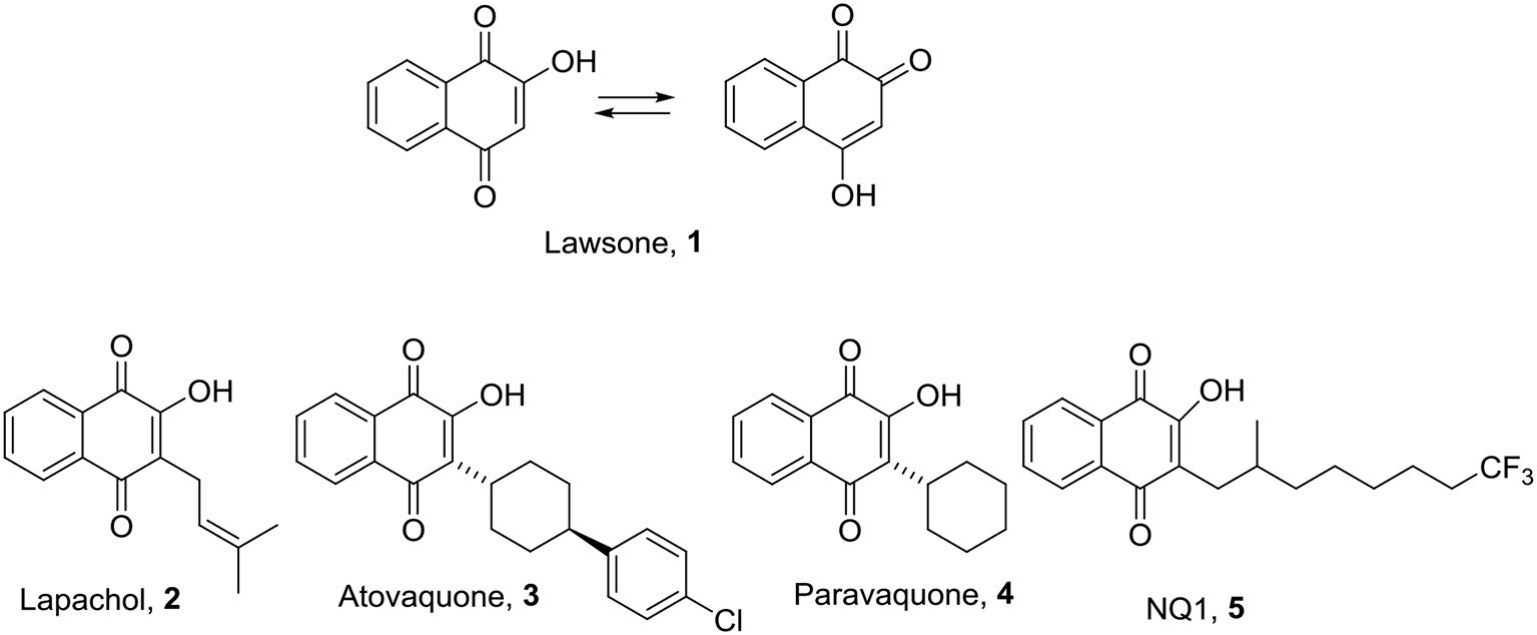
Lawsone and bioactive lawsone derivatives. NQ1: 2-OH-3-(2-methyl-trifluorooctyl)-naphthoquinone.

Lawsone [2- Hydroxy-1,4-naphthoquinone, (**1**)] or hennotannic acid, is a natural compound from the naphthoquinones family and can be obtained from leaves of henna (*Lawsonia* spp., family Lythraceae) as a red-orange pigment. The structure of Lawsone (**1**) has served for the development of new bioactive compounds and its scaffold is present in compounds with several valuable biological activities such as lapachol (**2**), atovaquone (**3**), parvaquone (**4**), and NQ1 (2-OH-3-(2-methyl-trifluorooctyl)-naphthoquinone) (**5**) (Figure 1) (Jordão et al., 2015). Lawsone (**1**) and its synthetic derivatives have shown promising biological results with antibacterial, antifungal, antitumor, and antiparasitic effects (Rahmoun et al., 2012; Barani et al., 2018; Al Nasr et al., 2019), their use, or study as antiplatelet agents have not been described, although the antiplatelet activity of other quinones has been reported (Fuentes et al., 2018). The current work aims to evaluate the antiplatelet effects of a series of lawsone thio-derivatives, which were obtained using on-water methodology, which was optimized by microwave irradiation for those cases that yielded poor results with conventional heating.

## Materials and Methods

### Chemicals, Reagents, General Procedures, and Apparatus

^1^H and ^13^C NMR spectra were obtained from a spectrometer operating at either 400.13 MHz (^1^H) or 100.61 MHz (^13^C). Measurements were carried out at 300°K. Chemical shifts are reported as ppm downfield from TMS for ^1^H NMR. ^13^C NMR spectra were recorded with the NMR spectrometer calibrated with CDCl_3_. All melting points are uncorrected and were determined using an Electrothermal 9100 apparatus. IR spectra (KBr discs) were recorded on an FT-IR spectrophotometer; wave numbers are reported in cm^−1^. High-resolution mass spectra were obtained on an orthogonal time-of-flight (TOF) mass spectrometer (QTOF Micro, Micromass UK) or a Q Exactive Focus (Thermo Scientific, USA). Silica gel 60 (230–400 mesh ASTM) and TLC aluminum sheets silica gel 60 F254 were used for flash-column chromatography and analytical TLC, respectively (Urra et al., 2016; Mendez et al., 2020).

### Synthesis of Compounds

#### General Procedure Using Conventional Heating

A mixture of 2.87 mmol of lawsone and 1.44 mmol of the respective thiol in 15 mL of water was heated at 50°C overnight, and then the mixture was extracted with ethyl acetate (30 mL × 3) the organic phase was dried with anhydrous sodium sulfate and then evaporated under a vacuum.

#### General Procedure With Microwave Irradiation

In a 100 mL ACE pressure tube with a magnetic stir bar, a mixture of 1.44 mmol of lawsone and 0.72 mmol of the respective thiol in water (6 mL) was heated at 50°C for 20 min under microwave irradiation (CEM Discover, Matthews, NC, USA). Then the mixture was extracted with ethyl acetate (30 mL × 3), and the organic phase dried with anhydrous sodium sulfate and evaporated under a vacuum (Tandon and Maurya, 2009; Tandon et al., 2010). Afterward, lawsone thio-derivatives **1**–**8**, were purified by flash chromatography with ethyl acetate:hexane:methanol 5:2:2 (Table 1).

**Table 1:** Synthesis of lawsone thio-derivatives.

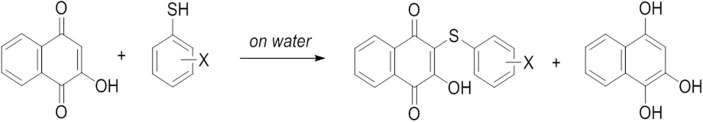			
**Compound**	**X**	**Yield (%)**	
		**Conventional heating**	**Microwave irradiation**
**1**	H	64	98
**2**	*o*-Br	26	36
**3**	*m*-Br	34	71
**4**	*p*-Br	8	18
**5**	*o*-CO_2_Me	8	20
**6**	*o*-F	84	–
**7**	*m*-F	84	–
**8**	*p*-F	86	–

#### 2-Hydroxy-3-(phenylthio)naphthalene-1,4-dione (1) (Tandon and Maurya, 2009)

By reaction of lawsone with benzenethiol compound **1** was obtained. ^1^H NMR δ (DMSO-d6): 7.2-7.3 (m, 5H), 7.9 (d, *J* = 7.3 Hz, 1H), 8.0 (t, *J* = 7.3 Hz, 2H), 8.1 (t, *J* = 7.4 Hz, 1H).

#### 2-((2-bromophenyl)thio)-3-hydroxynaphthalene-1,4-dione (2)

By reaction of lawsone with 2-bromobenzenethiol, compound **2** was obtained: red solid; mp 270.8 (d); ^1^H NMR δ (DMSO-d6): 6.71 (t, *J* = 7.4 Hz, 1H), 6.78 (d, *J* = 7.6 Hz, 1H), 6.86 (t, *J* = 7.4 Hz, 1H), 7.28 (d, *J* = 7.9 Hz, 1H), 7.49 (t, *J* = 7.4 Hz, 1H), 7.65 (t, *J* = 7.6 Hz, 1H), 7.86 (d, *J* = 7.6 Hz, 1H), 8.05 (d, *J* = 7.6 Hz, 1H); ^13^C NMR δ (DMSO-d6): 105.51, 122.32, 124.45, 126.28, 126.41, 126.66, 127.64, 129.87, 131.19, 134.60, 136.05, 143.29, 172.94, 180.09, 186.86; IR (KBr, cm^−1^): 3426, 2919, 2850, 1712, 1656, 1646, 1583, 1531, 1276, 744; HRMS (ESI) m/z calculated. for C_16_H_9_BrO_3_S M^+^: 359.9456, found: 359.9459.

#### 2-((3-bromophenyl)thio)-3-hydroxynaphthalene-1,4-dione (3)

By reaction of lawsone with 3-bromobenzenethiol, compound **3** was obtained: red solid; mp 231.3°C (d); ^1^H NMR δ (DMSO-d6): 6.92 (t, *J* = 7.8 Hz, 1H), 6.99 (ddd, *J* = 8.1 Hz, *J*_2_ = *J*_3_ = 1.6 Hz, 1H), 7.02 (ddd *J*_1_ = 7.6Hz, *J*_2_ = *J*_3_ = 1.2 Hz, 1H), 7.10 (dd, *J*_1_ = *J*_2_ = 1.6 Hz, 1H), 7.52 (td, *J*_1_ = 7.4 Hz *J*_2_ = 1 Hz, 1H), 7.65 (td, *J*_1_ = 7.6 Hz *J*_2_ = 1 Hz, 1H), 7.76 (s,1H), 7.89 (d, *J* = 7.6 Hz, 1H), 8.04 (dd, *J*_1_ = 7.6 Hz, *J*_2_ = 1 Hz, 1H); ^13^C NMR δ (DMSO-d6): 107.34, 116.94, 126.41, 126.94, 127.65, 130.57, 131.12, 131.46, 135.12, 135.72, 138.99, 180.75, 188.11; IR (KBr, cm^−1^): 3428, 2919, 2850, 1664, 1587, 1529, 1276, 738; HRMS (ESI) m/z calculated. for C_16_H_9_BrO_3_S M^+^: 359.9456, found: 359.9474.

#### 2-((4-Bromophenyl)thio)-3-hydroxynaphthalene-1,4-dione (4)

By reaction of lawsone with 4-bromobenzenethiol, compound **4** was obtained: red solid; mp 271.2°C (d); ^1^H RMN δ (DMSO-d6): 6.95 (d, *J* = 8.2 Hz, 2H), 7.08 (d, *J* = 8.2 Hz, 2H), 7.50 (t, *J* = 7.4 Hz, 1H), 7.53 (s, 1H), 7.66 (t, *J* = 7.5 Hz, 1H), 7.86 (d, *J* = 7.4 Hz, 1H), 8.04 (d, *J* = 7.6 Hz, 1H); ^13^C RMN δ (DMSO-d6): 116.95, 126.41, 126.95, 127.66, 130.57, 131.12, 131.46, 135.12, 135.71, 138.93, 180.77, 188.14; IR (KBr, cm^−1^): 3430, 2919, 2850, 1643, 1585, 1536, 1274, 740; HRMS (ESI) m/z calcd. for C_16_H_9_BrO_3_S M^+^: 359.9456, found: 359.9479.

#### Methyl 2-((3-hydroxy-1,4-dioxo-1,4-dihydronaphthalen-2-yl)thio)benzoate (5)

By reaction of lawsone with methyl thiosalicylate, compound **5** was obtained: red solid; mp 235°C (d); ^1^H NMR δ (DMSO-d6): 3.84 (s, 3H, CH_3_), 6.95 (t, *J* = 7.4 Hz, 1H), 7.04 (d, *J* = 8.1 Hz, 1H), 7.15 (t, *J* = 7.4, 1H), 7.57 (t, *J* = 7.5 Hz, 1H), 7.70 (t, *J* = 7.5 Hz, 1H), 7.80(d, *J* = 7.8 Hz, 1H), 7.91(d, *J* = 7.6 Hz, 1H), 8.00(d, *J* = 7.6 Hz, 1H), 8.07(s, 1H);^13^C NMR δ (DMSO-d6): 51.94, 105.71, 122.66, 126.13, 126.15, 126.17, 126.52, 130.94, 131.20, 131.47, 134.49, 136.27, 144.90, 166.70, 173.26, 179.86, 186.42; IR (KBr, cm^−1^): 3428, 2919, 2850, 1706, 1662, 1587, 1531, 1276, 1251; HRMS (ESI) m/z calcd. for C_18_H_12_O_5_S: 340.0405, found: 340.0392.

#### 2-((2-Fluorophenyl)thio)-3-hydroxynaphthalene-1,4-dione (6)

By reaction of lawsone with 2-fluorobenzenethiol, compound **6** was obtained: red solid; mp 260.8°C (d); ^1^H NMR δ (DMSO-d6): 6.61–6.64 (m, 1H), 6.70-6.80 (m, 2H), 6.84 (t, *J* = 7.5 Hz, 1H), 7.43-7.51 (m, 1H), 7.62 (t, *J* = 7.5 Hz, 1H), 7.84 (d, *J* = 7.5 Hz, 1H), 8.03(d, J = 7.5 Hz, 1 H); ^13^C NMR δ (DMSO-d6): 105.00, 114.62 (d, *J* = 21.8 Hz), 124.05 (d, *J* = 0.9 Hz), 125.08 (d, *J* = 7.3 Hz), 126.39, 126.47, 126.70, 127.68, 130.93, 131.21, 134.68, 135.80, 159.22 (d, *J* = 241.2 Hz), 172.96, 180.70, 187.31; IR (KBr, cm^−1^): 3428, 2917, 2850, 1673, 1587, 1533, 1469, 1276, 1213; HRMS (ESI) m/z calcd. for C_16_H_9_BrO_3_S: 300.0256, found: 300.0270.

#### 2-((3-Fluorophenyl)thio)-3-hydroxynaphthalene-1,4-dione (7)

By reaction of lawsone with 3-fluorobenzenethiol, compound **7** was obtained: red solid; mp 268.7°C(d); ^1^H NMR δ (DMSO-d6): 6.50-6.77(m, 2H), 6.82 (t, *J* = 7.0 Hz, 1H), 7.34 (s, 1H), 7.46(t, *J* = 6.9 Hz, 1H), 7.63 (t, *J* = 7.3 Hz, 1H), 7.84 (d, *J* = 7.0Hz, 1H), 8.04 (d, *J* = 7.3 Hz, 1H); ^13^C NMR δ (DMSO-d6): 102.26, 114.87 (d, *J* = 21.1 Hz), 124.55 (d, *J* = 2.9 Hz), 124.69 (d, *J* = 7.3 Hz), 126.21, 126.45, 127.14 (d, *J* = 2.9 Hz), 127.95 (d, *J* = 16 Hz), 131.37, 131.61, 134.75, 136.35, 158.92 (d, *J* = 239.8 Hz), 173.29, 179.13, 185.73; IR (KBr, cm^−1^): 3430, 2360, 2339, 1664, 1587, 1533, 1469, 1278, 1222; HRMS (ESI) m/z calculated. for C_16_H_9_FO_3_S: 300.0256, found: 300.0233.

#### 2-((4-Fluorophenyl)thio)-3-hydroxynaphthalene-1,4-dione (8)

By reaction of lawsone with 4-fluorobenzenethiol, compound **8** was obtained: red solid; mp 268.0°C (d); ^1^H NMR δ (DMSO-d6): 6.76 (t, *J* = 8.6 Hz, 2H), 7.03 (dd, *J*_1_ = 8 Hz, *J*_2_ = 5.5 Hz, 1H), 7.50(t, *J* = 7.4 Hz, 1H), 7.63(t, *J* = 7.5 Hz, 1H), 7.79(s, 1H), 7.88(d, *J* = 7.6 Hz, 1H), 8.0(d, *J* = 7.6 Hz, 1H); ^13^C NMR δ (DMSO-d6): 106.91, 115.14, (d, *J* = 21.80 Hz), 126.09, 126.54, 127.40 (d, *J* = 7.3 HZ), 130.98, 131.29 (d, *J* = 13.8 Hz), 134.32, 135.51, 136.18, 159.98 (d, *J* = 241.2 Hz), 172.83, 179.97, 186.60; IR (KBr, cm^−1^): 3424, 2919, 2850, 2358, 1661, 1587, 1526, 1381, 1273, 1221; HRMS (ESI) m/z calculated. for C_16_H_9_FO_3_S: 300.0256, found: 300.0262.

### Human Washed Platelets

Platelets were obtained from citrated whole blood of healthy volunteers (two weeks drugs-free) with written informed consent, as previously described (Fuentes et al., 2014a).

### Lactate Dehydrogenase (LDH)-Based Cytotoxicity Assay

Washed platelets (200 × 10^9^ platelets/L) were incubated for 10 min at 37°C with DMSO (vehicle) 0.2% or 100 μM of compounds. Then, platelets are centrifuged (800 × g for 8 min), and 50 μL aliquots of the resulting supernatant analyzed by the LDH assay (Cayman Chemical, USA). The reported results correspond to the percentage of total enzyme activity from a control incubation lysed with 0.3% Triton X-100 (Kim et al., 2009; Mendez et al., 2020).

### Platelet Aggregation

Platelet aggregation was evaluated using a lumi-aggregometer (Chrono-Log, Haverton, PA, USA) and monitored by light transmission according to Fuentes et al. (Fuentes et al., 2014a; Mendez et al., 2020). Briefly, washed platelets (200 × 10^9^ platelets/L) were pre-incubated for 5 min at 37°C and pH 7.4 with vehicle or different doses of compounds; then platelet aggregation was induced by TRAP-6 (5 μM) or collagen (1 μg/mL) and measured for 5 min at 37°C while stirring.

### Flow Cytometry Study

Externalization of phosphatidylserine, glycoprotein (GP) IIb/IIIa activation, P-selectin expression, CD63 release, and ROS levels on platelets were determined by flow cytometry (Ritchie et al., 2000; Fuentes et al., 2014b; Walsh et al., 2014). All results were analyzed in the Accuri C6 flow cytometer (BD, Biosciences, USA).

### Statistical Analysis

The data obtained were presented as a mean ± standard deviation (*SD*) of three independent experiments and analyzed using Prism 8.3 software (GraphPad Inc., San Diego CA, USA). The half-maximal inhibitory concentration (IC_50_) was calculated from dose-response curves. Differences between samples were analyzed using Tukey or paired t-test (Vaiyapuri et al., 2013). *P*-values < 0.05 were considered as significant (Fuentes et al., 2014b; Munoz-Gutierrez et al., 2017).

## Results and Discussion

### Synthesis

Several attempts to reproduce the reported yield in the reaction of unsubstituted benzenethiol with lawsone were unsuccessful, even raising the described time to overnight. However, the reaction with fluoro-substituted benzenethiols gave good yields, contrary to Bromo substituted derivatives which gave very low yields. These results show a correlation between yields of the reaction and the solubility of benzenethiols in water. To improve the low yields obtained with the less soluble thiols, microwave irradiation, instead of conventional heating was used, in this way better yields and very reduced reaction times were obtained. The structural assignment of the pure new products relied on the disappearing of the quinone proton of lawsone and the signals reported by the aromatic ring of the thiols, besides high-resolution mass spectrometry. ^1^H and ^13^C NMR spectrum of each compound were included as Supplementary Information.

### Cytotoxicity Activity on Platelets

Before analyzing the potential antiplatelet activity of the synthesized compounds, we evaluated the cytotoxic activity that lawsone and its derivative may have on platelets. After incubating platelets for 10 min at 37°C with each compound, cytotoxicity detection by LDH leakage showed that compounds **1**, **2**, **4**, **6**, **7**, and **8** at the higher concentration tested (100 μM) did not exert cytotoxicity on platelets (Table 2). Quinones represent a class of organic compounds that have previously shown that through quite complex mechanisms can have certain levels of cytotoxicity (Bolton et al., 2000; Bolton and Dunlap, 2017). Quinone-based compounds have been regarded as among the most troublesome of all pan-assay interference compounds (PAINS) chemotypes, limiting their medical potential (Baell and Nissink, 2018; Qin et al., 2018). Alternatively, quinones are highly redox-active molecules which can generate oxidative damage in intracellular macromolecules such as lipids, proteins, and DNA (Bolton et al., 2000). In this study, we showed that lawsone and compounds **3** and **5** had a cytotoxic activity with an increase of LDH released by damaged platelets.

**Table 2:** Cytotoxic activity of thio-derivatives of 2-hydroxy-1,4-naphthoquinone (lawsone) on washed platelets.

**Compound**	**Cytotoxic activity**
	**Mean (%) ± *SD***
Lawsone	32.80 ± 3.13^**^
1	22.35 ± 2.94
2	14.72 ± 10.92
3	36.51 ± 4.23^***^
4	22.38 ± 5.78
5	31.12 ± 3.06^**^
6	21.77 ± 2.37
7	21.50 ± 2.37
8	21.62 ± 4.17
Vehicle (DMSO 0.2%)	12.49 ± 2.69

** p < 0.01 and

*** *p < 0.001 analyzed by Tukey test*.

### Antiplatelet Activity of New Thio-Derivatives of 2-Hydroxy-1,4-Naphthoquinone

Antiplatelet therapy is called the treatment for the prevention of platelet activation-associated thrombosis (Harrington et al., 2003). Although aspirin and clopidogrel have been the cornerstone in the management of platelet regulation, a high number of patients continued suffering recurrent thrombotic events (Fox et al., 2004; Price et al., 2011). Since the available therapeutic drugs to control non-desired platelet activation remains limited, the search for new antiplatelet agents is necessary (Mousa, 1999; Moura et al., 2016). In this regard, some structure-activity relationship studies with quinone derivatives have shown that the variability of inhibition of platelet activation by these type of compounds is governed by the nature of the substituent (Kim et al., 2001; Fuentes et al., 2018), as explained below: (i) The oxidation of the B-ring of hydroxyl-methoxyisoflavone to methoxyflavone quinone, and the conversion of flavone and isoflavones into their corresponding flavanquinone or isoflavanquinones, result in an increase and potent antiplatelet activity (Chang et al., 2000). (ii) The inhibition of platelet aggregation by 2-alkoxy-1,4-naphthoquinone increases with 2-alkoxy chain length (Lien et al., 2002). (iii) The inclusion of a free amino group in the thiosulfonate moiety of quinone (4-aminobenzenesulfonothioic acid S-[(9,10-dihydro-1,4-dimethoxy-9,10-dioxo2-anthracenyl)methyl] ester) increased its antiplatelet activity (Bolibrukh et al., 2015), (iv) The inhibition of platelet activation by alpha-tocopherol quinone is greater than that of vitamin E (Rao et al., 1981), and (v) *gem*-diethyl/methyl substitutions and the addition/modifications of the third ring of *ortho*-carbonyl hydroquinone scaffold influence on the selective index (IC_50_ TRAP-6/IC_50_ Collagen) and the inhibitory capacity of platelet aggregation (Mendez et al., 2020).

Lawsone is a natural naphthoquinone that is easily obtained from natural sources and is currently commercially available in bulk. It has been used as the starting material for the synthesis of a variety of biologically active compounds and materials with interesting properties in fungi, bacteria, and viruses (Jordão et al., 2015). The synthesis reaction from lawsone described in this article has allowed the development of a variety of lawsone thio-derivatives that could be potentially useful for the treatment of platelets aggregation-associated thrombosis diseases. In this way, we observed that the platelet antiaggregant activity depends on the nature and position of the substituent at the phenyl moiety (Table 3). All tested compounds showed higher effectiveness (lower IC_50_ values) against platelet aggregation induced by collagen than TRAP-6 (Table 3). It has described that given the variability of quinone in biological activities it is likely that there is a low selectivity in its antiplatelet activity (Ma and Long, 2014). Our work, as shown in Table 3, shows that compound **6** presents a specific inhibition of collagen-induced platelet aggregation. Atherothrombosis is initiated by collagen exposure from endothelial damage to circulating platelets. These platelets, via the GPVI receptor, bind directly to collagen triggering platelet activation (Nieswandt and Watson, 2003). In this context, our results suggest that pretreatment of platelets with compound **6** down-regulates the GPVI-mediated signaling pathway (Jung et al., 2017).

**Figure 2:**
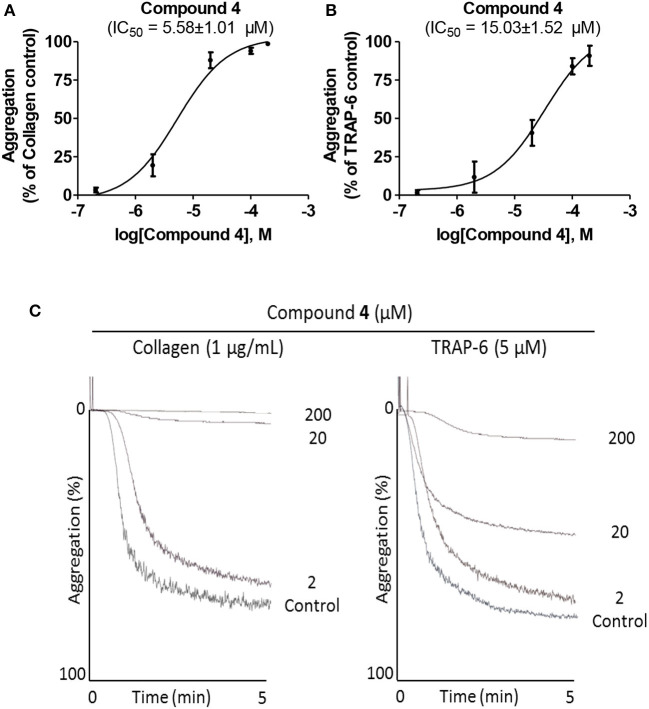
Inhibition of platelet aggregation by compound **4**. **(A)** and **(B)** IC_50_ of compound **4** was determined from the dose-response curves. **(C)** Representative curves for platelet aggregation pre-incubated with compound **4** at 2, 20, and 200 μM, and induced by collagen (1 μg/mL) or TRAP-6 (5 μM).

**Figure 3:**
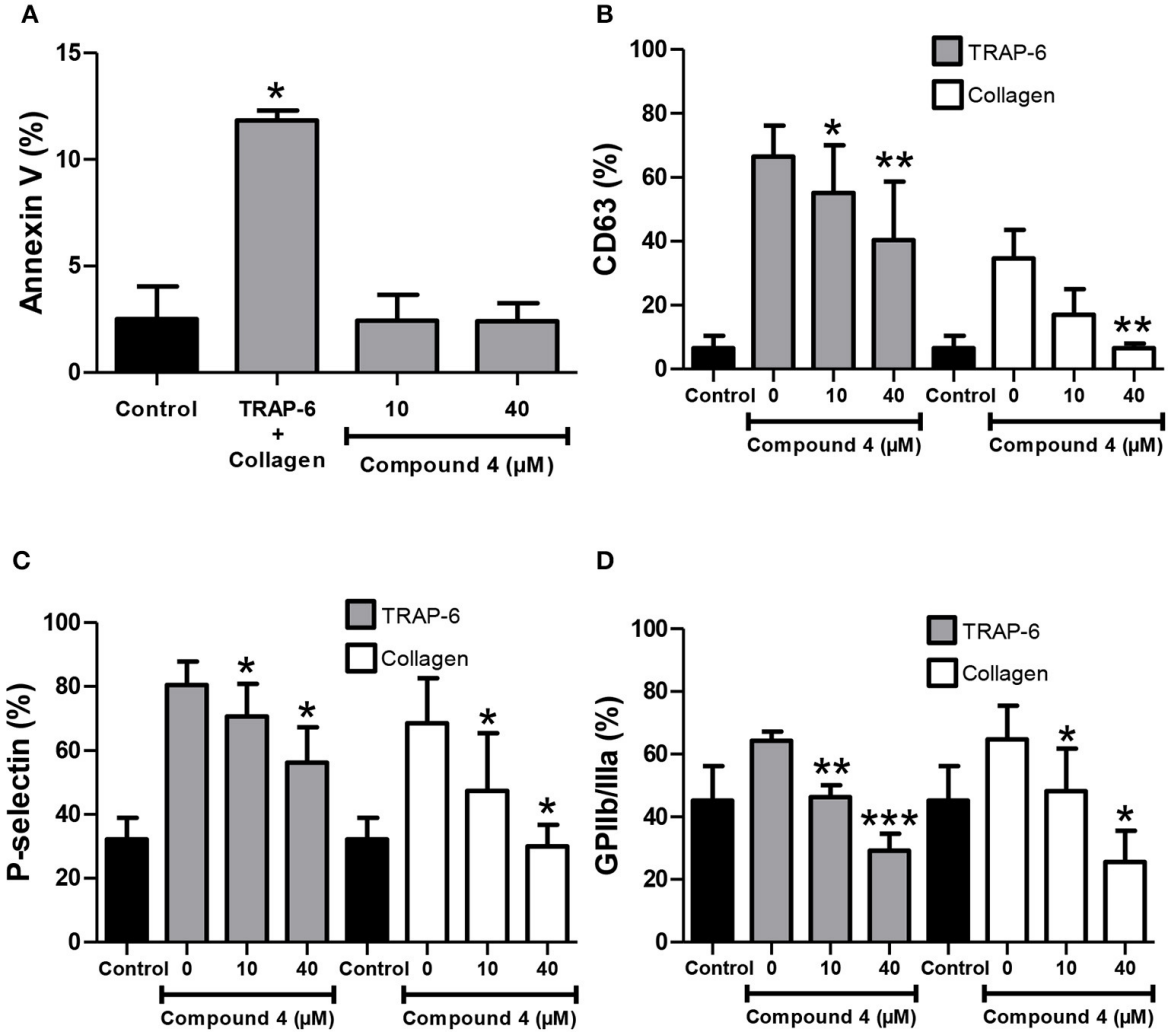
Antiplatelet activity of compound **4**. **(A)** Externalization of phosphatidylserine was evaluated by annexin-V binding. **(B)** Levels of CD63 on the surface of platelets after activation by TRAP-6 (5 μM) or collagen (1 μg/mL). **(C)** P-selectin expression was measured by flow cytometry in platelets stimulated with TRAP-6 (5 μM) or collagen (1 μg/mL). **(D)** GPIIb/IIIa activation stimulated by TRAP-6 (5 μM) or collagen (1 μg/mL) was determined by flow cytometry using the PAC-1 antibody. The graph depicts the mean ± *SD* of *n* = 3 experiments. The black graph is the control of resting platelets. ^*^p < 0.05, ^**^p < 0.01, and ^***^p < 0.001 vs. 0 (vehicle DMSO 0.2%).

**Table 3:** Inhibition of platelet aggregation by thio-derivatives of 2-hydroxy-1,4-naphthoquinone (lawsone) on TRAP-6- or collagen-stimulated platelets.

**Compound**	**IC**_****50****_ **(μM)** **±** ***SD***	
	**TRAP-6 (5 μM)**	**Collagen (1 μg/mL)**
1	155.40 ± 55.20	14.58 ± 5.43
2	111.63 ± 17.53	11.17 ± 3.90
4	15.03 ± 1.52	5.58 ± 1.01
6	>500	10.59 ± 2.25
7	134.16 ± 17.34	11.13 ± 1.69
8	107.56 ± 52.98	12.05 ± 1.21

Small structural changes led to the synthesis of non-toxic lawsone thio-derivatives with potent antiplatelet activity (Tables 1, 3). Our experimental design led to the synthesis of antiplatelets compounds, being compound **4** the most potent among the non-toxic compounds. Compound **4** presents the lower IC_50_ values, being 15.03 ± 1.52 μM for TRAP-6, and 5.58 ± 1.01μM for collagen respectively (Figure 2). Thus, we decided to deeply analyze the effects of compound **4** in platelets. In addition to its non-toxic activity (Table 2), we demonstrated that compound **4** was unable to affect phosphatidylserine exposure (Figure 3A), a modification associated with platelet mitochondrial apoptotic-like events (Augereau et al., 2004). In non-activated platelets, CD63 is present on the dense granules and exposed in membranes after platelet activation (Israels and McMillan-Ward, 2005). CD63 is essential for P-selectin function, which is a thrombo-inflammatory molecule that participates in platelet activation and aggregation (Théorêt et al., 2011). As shown in Figure 3B, compound **4** decreased the expression of CD63 on platelets' membrane after activation with either TRAP-6 or collagen. As expected, platelets' P-selectin expression on activated platelets by TRAP-6 or collagen was inhibited by compound **4** at 10 and 40 μM (Figure 3C). In activated platelets, GPIIb/IIIa is very important at the final step of platelet activation leading to aggregation (Chen et al., 2015). We analyzed the activated form of GPIIb/IIIa by studying the binding of FITC-labeled PAC-1 antibody by flow cytometry (Fuentes et al., 2014a). Figure 3D demonstrated that compound **4** inhibited TRAP-6- and collagen-induced PAC-1 binding to platelets at compound levels similar to those that inhibited platelet aggregation (Table 3).

### Limitation and Perspectives

The set of experiments presented were designed to demonstrate that thio-derivatives of lawsone are useful novel scaffolds to synthesize antiplatelet compounds. The small number of compounds comprising the series analyzed was limited in most part due to the difficulty in accessing human platelets from a significant number of donors. Besides this fact, we were able to determine that the modification inserted for compound **4** synthesis improved the activity on platelet aggregation. Future studies will be directed toward increasing the series of lawsone-derivatives and comparing their effects in different biological models. Although it was not in the scope of the current work, the lack of testing of cytotoxicity, bio-disponibility, and mechanisms of action on *in vivo* systems decrease the impact of compound **4** for pharmacological purposes. However, these will be solved in the future with studies in cells and animal models at different pathophysiological conditions.

In conclusion, our study revealed that thio-derivatives of lawsone are useful novel scaffolds to synthesize antiplatelet compounds, with compound **4** being the most potent.

## Data Availability Statement

The raw data supporting the conclusions of this article will be made available by the authors, without undue reservation.

## Ethics Statement

The studies involving human participants were reviewed and approved by Scientific ethics committee, Universidad de Talca. The patients/participants provided their written informed consent to participate in this study.

## Author Contributions

EF, RA-M, and MM-Ci: conceptualization. MM-Cá and DM: formal analysis. EF, and RA-M: writing—original draft preparation and funding acquisition. AT: writing—review and editing. All authors contributed to the article and approved the submitted version.

## Conflict of Interest

The authors declare that the research was conducted in the absence of any commercial or financial relationships that could be construed as a potential conflict of interest.
