# Supplementary material for: Synthesis and Biological Evaluation of Thio-Derivatives of 2-Hydroxy-1,4-Naphthoquinone (Lawsone) as Novel Antiplatelet Agents
Source: Front Chem. 2020 Aug 4;8:533. doi: 10.3389/fchem.2020.00533 (PMC7417813; doi:10.3389/fchem.2020.00533)
Supplement: Supplementary file 1 [file Table_1.DOCX]

**Supplementary information**

**SYNTHESIS AND BIOLOGICAL EVALUATION OF THIO-DERIVATIVES OF 2-HYDROXY-1,4-NAPHTHOQUINONE (LAWSONE) AS NOVEL ANTIPLATELET AGENTS**

Matías Monroy-Cárdenas, Diego Méndez, Andrés Trostchansky, Maximiliano Martínez-Cifuentes, Ramiro Araya-Maturana, Eduardo Fuentes

2-((2-bromophenyl)thio)-3-hydroxynaphthalene-1,4-dione (**2**)

2-((3-bromophenyl)thio)-3-hydroxynaphthalene-1,4-dione (**3**)

2-((4-bromophenyl)thio)-3-hydroxynaphthalene-1,4-dione (**4**)

methyl 2-((3-hydroxy-1,4-dioxo-1,4-dihydronaphthalen-2-yl)thio)benzoate (**5**)

2-((2-fluorophenyl)thio)-3-hydroxynaphthalene-1,4-dione (**6**)

2-((3-fluorophenyl)thio)-3-hydroxynaphthalene-1,4-dione (**7**)

2-((4-fluorophenyl)thio)-3-hydroxynaphthalene-1,4-dione (**8**)
